# Complete Transcriptome Profiling of Normal and Age-Related Macular Degeneration Eye Tissues Reveals Dysregulation of Anti-Sense Transcription

**DOI:** 10.1038/s41598-018-21104-7

**Published:** 2018-02-14

**Authors:** Eun Ji Kim, Gregory R. Grant, Anita S. Bowman, Naqi Haider, Harini V. Gudiseva, Venkata Ramana Murthy Chavali

**Affiliations:** 1 Institute for Translational Medicine and Therapeutics, University of Pennsylvania School of Medicine, Philadelphia, Pennsylvania USA; 2 Department of Genetics, University of Pennsylvania, Philadelphia, Pennsylvania USA; 3 Department of Ophthalmology, University of Pennsylvania School of Medicine, Philadelphia, Pennsylvania USA; 4 Functional Genomics Lab, University of Pennsylvania, Philadelphia, Pennsylvania USA

## Abstract

Age-related macular degeneration (AMD) predominantly affects the retina and retinal pigment epithelium in the posterior eye. While there are numerous studies investigating the non-coding transcriptome of retina and RPE, few significant differences between AMD and normal tissues have been reported. Strand specific RNA sequencing of both peripheral retina (PR) and RPE-Choroid-Sclera (PRCS), in both AMD and matched normal controls were generated. The transcriptome analysis reveals a highly significant and consistent impact on anti-sense transcription as well as moderate changes in the regulation of non-coding (sense) RNA. Hundreds of genes that do not express anti-sense transcripts in normal PR and PRCS demonstrate significant anti-sense expression in AMD in all patient samples. Several pathways are highly enriched in the upregulated anti-sense transcripts—in particular the EIF2 signaling pathway. These results call for a deeper exploration into anti-sense and noncoding RNA regulation in AMD and their potential as therapeutic targets.

## Introduction

Age-related macular degeneration (AMD) is the third largest cause of vision loss worldwide^1^. It is a progressive retinal disorder that involves loss of central vision, hypo- and hyper-pigmentation of the RPE, deposition of drusen in the Bruch’s membrane, and loss of photoreceptors, especially in the 8^th^ or 9^th^ decade^2–4^. The most severe visual loss due to AMD occurs when the disease progresses to one of the two advanced forms: dry (atrophic) AMD or wet (exudative or neovascular) AMD. Genome-Wide Association studies (GWAS) have associated certain mutations/variations in the genes involved in various biological pathways with onset, progression, and involvement of different stages of AMD^5–11^. Associated pathways include immune system, cholesterol metabolism, collagen/extra-cellular matrix processing and angiogenesis. Most of the known variations occur in the protein coding regions of genes, which comprise a very small percentage of the entire human genome^12^. With the development of next-generation sequencing technologies, it has become increasingly apparent that a greater part of the genome encodes for non-coding RNAs (ncRNAs). The ncRNA in eukaryotes probably exceed the total number of protein-coding genes^13^.

Transcriptome studies have generated significant interest in the role of ncRNAs in the maintenance of cellular processes and function. Based on their length, ncRNAs are broadly divided into short ncRNAs (<200 nucleotides: e.g. ribosomal RNA (rRNA)), small interfering RNA (siRNA), micro RNA (miRNA), small nuclear RNA (snRNA), small nucleolar RNA (snoRNA), piwi interacting RNA (piRNA) and long ncRNA (lncRNA, >200 nucleotides)^14^. They may be anti-sense, intergenic, interleaved, or overlapping with protein-coding genes^15–17^. In particular, their ability to base pair with other transcripts suggests they may be responsible for a variety of regulatory functions^18^. Transcriptome studies over the last two decades analyzed the posterior region of the eye using SAGE, microarrays and RNA Sequencing methodologies^19–23^. However, none of these studies have specifically addressed the differences in the transcriptome expression between the normal and AMD retinal tissues.

The posterior part of the eye consists of three layers; the neural retina, the RPE and the choroid. The RPE secretes a variety of growth factors to help maintain the structural integrity of choriocapillaris and photoreceptors. It also phagocytoses the photoreceptors and regulates ion and metabolic transport between the retina and choroid^24^. The macula is the cone rich, central part of the retina that is responsible for central vision and is affected in AMD. The retinal photoreceptors, the RPE, and the choroid, act in concert to maintain visual function; which makes these tissues natural targets for transcriptome studies of AMD. Gene expression in young and elderly human retinas was compared by Yoshida *et al*.^12^ using microarrays, indicating that the genes *KIAA0120*, *TRPIP1*, and *ISGF3G* were upregulated in younger retina^25^. Other microarray studies in young vs. old, and fetal vs. adult total retina or macular retina identified genes elevated in the fovea macula and peripheral retina^26,27^. Differentially expressed (DE) genes were identified in the macular retina, peripheral retina and in enriched RPE using the SAGE platform^28^. These studies indicate a spatial effect on gene expression^29^ and alternative transcription in these tissues^28^.

Here peripheral human retina (PR) and peripheral RPE-Choroid-Scleral (PRCS) tissues (from normal and AMD donors) were high throughput RNA sequenced to identify unknown transcripts, and quantify transcripts of coding and noncoding RNA that is not possible with microarrays or SAGE^23,30–32^. Unlike the PR samples, transcriptome analysis of the PRCS tissue layers were interrogated together due to the difficulty of separating individual layers in cadaver donor eye tissues without contamination. Access to high quality tissues and greater sequencing depth allowed for a robust transcriptome profiling which includes many non-coding species. Many unannotated non-coding genes exist in introns as anti-sense to the parent gene. Strand specific sequencing was employed to more easily identify such unannotated genes. Strand-specific sequencing also revealed a considerable amount of differential anti-sense transcription of protein coding genes with significant enrichment of several pathways. This high level of differential anti-sense expression suggests its potential functional and clinical relevance in AMD. In accordance with these findings, the focus of this paper is two-fold: an investigation of the non-coding RNA and anti-sense transcription in AMD as compared to normal.

A rapidly growing body of evidence points to the importance of noncoding RNA in normal and a wide variety of pathological processes. Our study is the first comprehensive transcriptome analysis of the total non-coding and anti-sense RNA profiles in the PR and PRCS tissues of normal and AMD donor eyes and the first to reveal that anti-sense RNAs may play an important role in the development and progression of AMD.

## Results

### Analysis of gene expression

**Figure 1:**
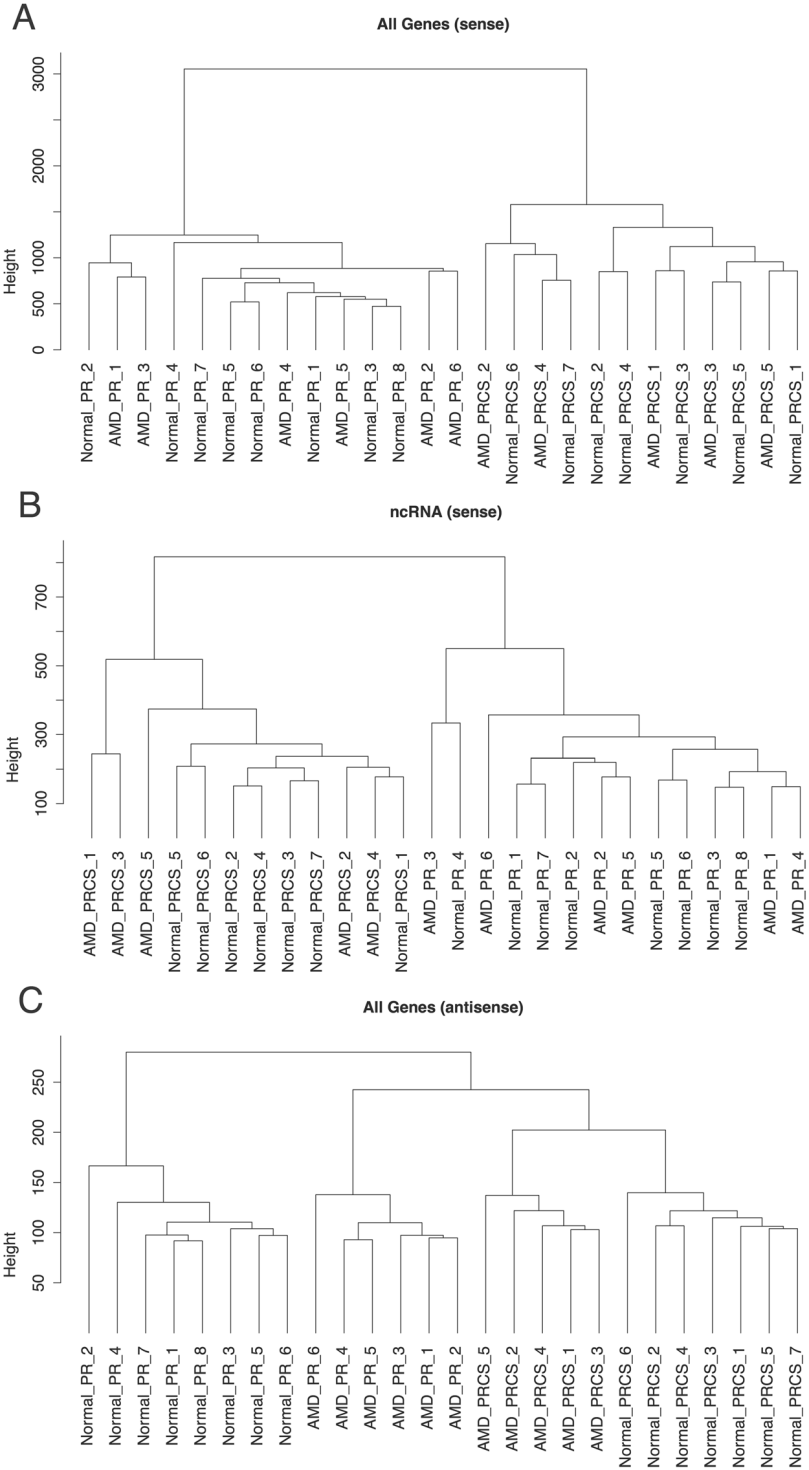
Hierarchical clustering of genes across 26 samples. (**A**) Clustering of sense gene expression, coding and noncoding. (**B**) Clustering of only noncoding genes. (**C**) Clustering of anti-sense gene expression.

Hierarchical clustering was performed for the sense and anti-sense gene expression; and was also performed using only the noncoding RNA (Fig. 1). A clear delineation between the PR and PRCS is observed between tissues, based on sense expression, for both coding and noncoding regions. The separation within AMD and normal tissue types, however, is not apparent. In contrast, using the anti-sense gene expression, a clear clustering of the samples is observed by their tissue type and disease status. We have analyzed all three-transcript types (gene-sense, gene-anti-sense and noncoding) in our study and the anti-sense signal is the most powerful indicator of disease state.

**Figure 2:**
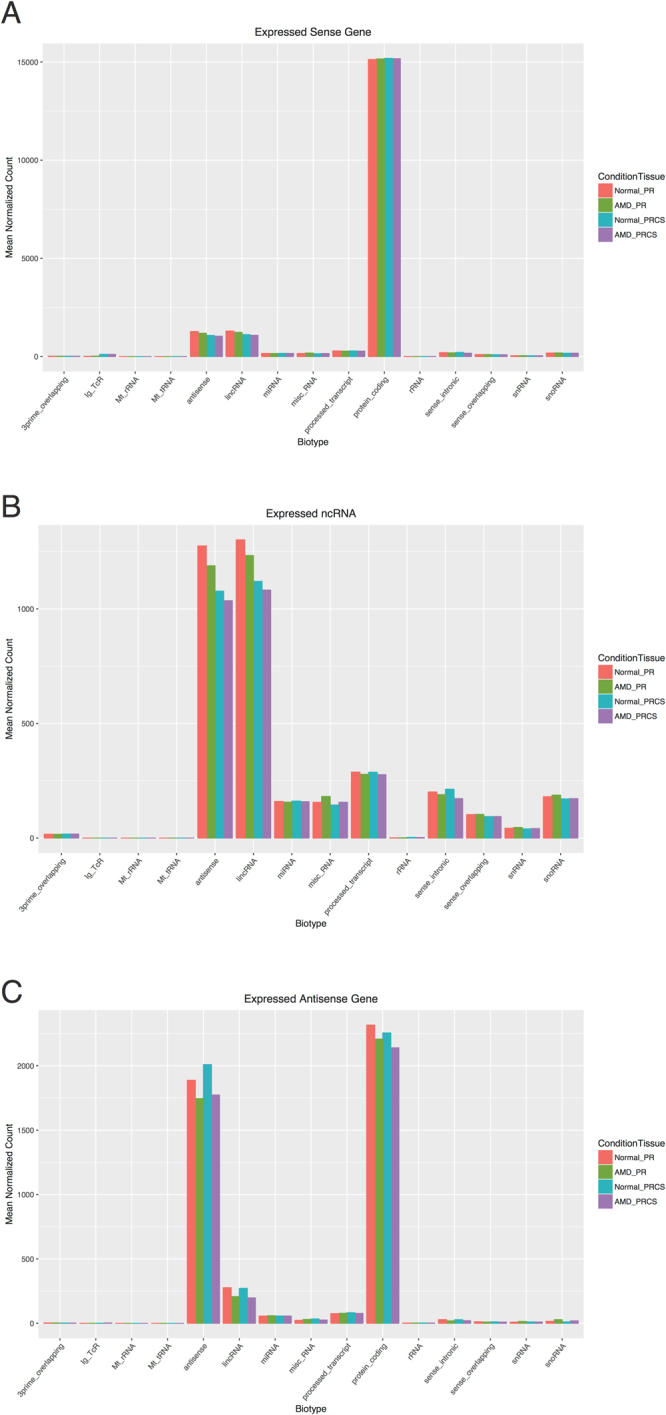
Distribution of biotypes of expressed transcripts in each category of biotypes is plotted by condition and tissue type. (**A**) All expressed sense transcripts. (**B**) Expressed ncRNA. (**C**) Expressed anti-sense transcripts.

Non-coding RNA was quantified and a minimum expression cutoff was defined by requiring a gene to have normalized average coverage ≥1. In total, 6,972 ncRNA were expressed in at least one sample using this criterion. The average number of expressed ncRNA is 3,582 in AMD-PR, 3,210 in AMD-PRCS, 3,725 in normal PR, and 3,330 in normal PRCS. The abundances of the various categories of noncoding RNA was compiled (Fig. 2B). The vast majority of expressed ncRNA comprised of anti-sense and lincRNA. The absence of the smaller noncoding types is due in part to the size selection in the library construction process. In both of the aforementioned categories however, the number of expressed transcripts is highest in Normal-PR. It should be noted that these categories are as defined by ENSEMBL and that “anti-sense” in this context refers to genes which exist in the introns of other genes (called the “parent” genes), and which are transcribed in the opposite direction of the parent. This is different from what will be referred to below as “anti-sense transcription” which refers to reads which map anti-sense to the *exons* of known genes.

**Figure 3:**
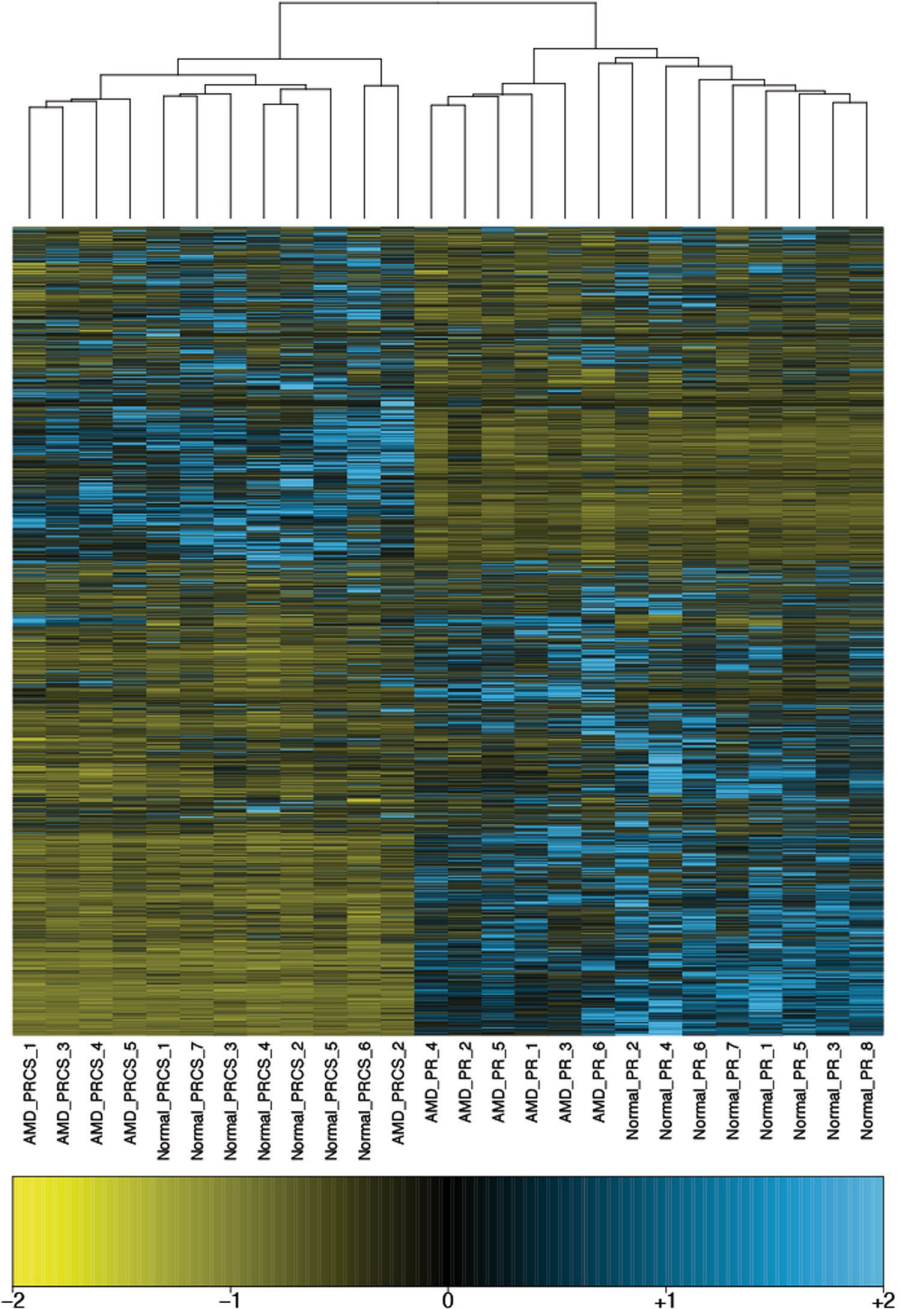
Expressed ncRNAs with high variability. Heatmap of expression in average coverage of the top 1000 most variably expressed ncRNAs, defined as those with the largest coefficient of variation across all tissue/disease types.

To explore the differences in ncRNA between the retina and PRCS tissue types and between normal and AMD eye tissues, hierarchical clustering was performed using the top 1,000 most variably expressed ncRNAs—defined as those with the largest coefficient of variation of normalized average coverage across all samples (Fig. 3). A clear delineation between the PR and PRCS samples was observed, indicating specialized signatures for transcripts between PR and PRCS in the normal and diseased states. Less apparent in ncRNA is the difference between normal and disease states.

### Differential anti-sense gene expression in AMD

**Figure 4:**
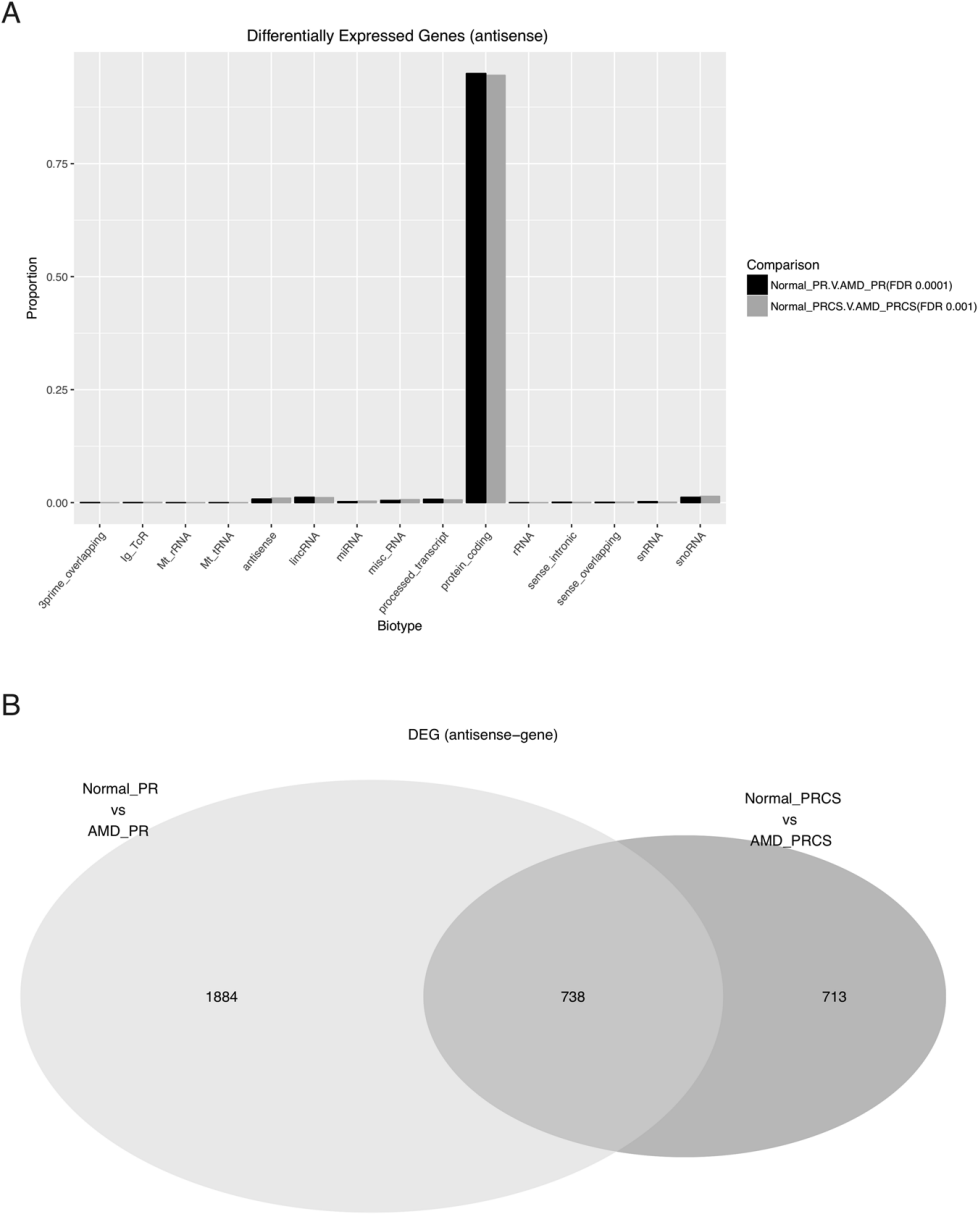
Differentially expressed antisense genes between normal and AMD comparisons. (**A**) Biotypes of differentially expressed antisense genes. (**B**) Venn diagram of differentially expressed antisense genes between PR and PRCS.

**Figure 5:**
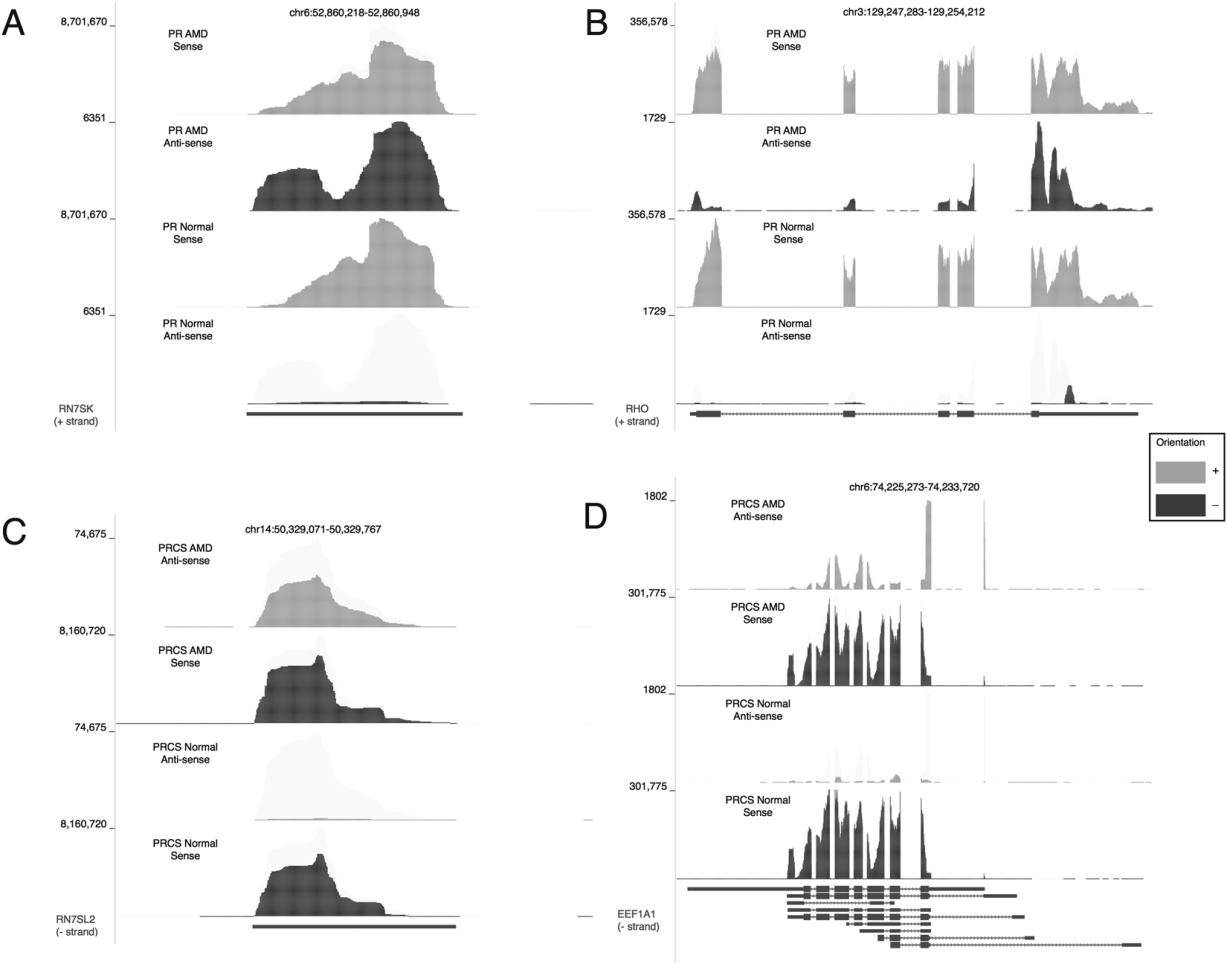
Differentially expressed anti-sense transcription. The coverage plots were generated merging normalized coverage of 5 replicates in each condition. The plus and minus tracks are displayed and scaled separately. Differentially expressed anti-sense expression is shown in (**A**) one exon gene RN7SK and (**B**) multi-exon gene RHO between AMD and Normal PR samples. For PRCS samples, (**C**) one exon gene RN7SL2 and (**D**) multi-exon gene EEF1A1 are shown as examples of differential anti-sense expression. Gene counts of individual samples for the four genes are available in Supplementary Table 5.

In contrast to the ncRNA results described above, significant differential expression of anti-sense genes transcripts is observed in the AMD-PR and AMD PRCS tissues when compared to the normal tissues. There are 2,025 anti-sense transcripts up-regulated and 597 transcripts down-regulated when normal PR was compared to the AMD PR (q = 0.0001). Comparison between normal and AMD PRCS resulted in 941 upregulated and 510 down regulated anti-sense genes that were differentially regulated (q = 0.001) (Table 1). The overwhelming majority of these genes are protein coding genes (Fig. 4). Figure 5 shows four typical examples, two in PR and two in PRCS. The single-exon gene RN7SK on Chromosome 6 is highly expressed in retina, as shown in the red depth-of-coverage plot (Fig. 5A). While there is a trace amount of anti-sense transcription of RN7SK in normal controls, as seen in the lower track (4^th^ track from the top), there is high anti-sense transcription of this gene in AMD (2^nd^ track from the top). This anti-sense signal represents reads that strictly align to the exons, they do not overlap introns and as seen in Fig. 5B they respect exon/exon junctions, albeit backwards. This is observed in both PR and PRCS tissues. In all cases the sense signal is higher than the anti-sense, and for the most part the sense signal is not differential. Complete genome browser tracks for this data are available at http://bit.ly/2zyL56k (merged by condition) and http://bit.ly/2Abl00W (all samples).

**Table 1:** The number of differentially expressed noncoding gene.

Comparison of disease states in tissues	Biotype	Number of DE transcripts
Normal PR vs AMD PR	Gene anti-sense	2622 (2025 up; 597 down)
Normal PRCS vs AMD PRCS	Gene anti-sense	1451 (941 up; 510 down)
Normal PR vs AMD PR	Gene sense	537 (211 up; 326 down)
Normal PRCS vs AMD PRCS	Gene sense	310 (176 up; 134 down)
Normal PR vs AMD PR	ncRNA	280 (170 up; 110 down)
Normal PRCS vs AMD PRCS	ncRNA	188 (136 up; 52 down)

Although there are hundreds of genes with down-regulated anti-sense transcription in AMD, they do not tend to target particular pathways. In contrast, among those genes with up-regulated anti-sense transcription in AMD in the retina, the EIF2 signaling pathway was predominant (enrichment *p*-value < 3.31E-26), suggesting a central role for this pathway and implicating ribosomal regulation. Other pathways that were significantly affected were regulation of elF4 and p70S6K signaling, mTOR signaling, phototransduction and mitochondrial dysfunction pathway. The anti-sense transcripts that were upregulated also appear to manage apoptosis pathways, mitochodrial function and NRF2 mediated oxidative stress response, which is known to contribute to retinal maintenance. The top networks identified include cell death and survival, cellular growth and proliferation and cellular assembly and organization as well as RNA posttranslational modification, repair and connective tissue disorders. The top relevant toxicology lists were mitochondrial dysfunction, NRF2-mediated oxidative stress response and PPAR/RXR RXR activation pathways. In addition to the pathways affected in the retina are the EIF2/mTOR, the differentially expressed anti-sense RNA in PRCS tissues on IPA analysis included those involved in clathrin and caveolar mediated endocytosis signaling, actin cytoskeleton signaling and RhoGDI signaling. Cellular function and maintenance, cellular development, cell death and survival were key molecular and cellular functions along with nervous system and development function, which are regulated in PRPC.

Among individual upregulated anti-sense transcripts in AMD retina as compared to normal, a five-fold higher expression of Transferrin in the AMD retinal tissue was observed (*q*-value = 9.68 E-16). Other protein coding genes up regulated in AMD include glyceraldehyde-3-phosphate dehydrogenase, nuclear ubiquitous casein and cyclin-dependent kinase substrate 1(NUCKS1), glutathione S-transferase alpha 4 (GSTA4) and interphotoreceptor matrix proteoglycan-1 (IMPG1), a gene encoding the Sialoprotein associated with cones and rods (SPACR)^33^. The expression of many small nuclear pseudogenes and small nucleolar RNA such as SNORD3A and SNORA73B appear to be upregulated in AMD, implying an effect on the global regulation of the transcription machinery that is necessary for normal retinal maintenance (Supplementary Table 2). The lincRNA RMRP (RNA component of mitochondrial RNA processing endoribonuclease) is the predominant lincRNA that is significantly overexpressed in the AMD retina when compared to the normal tissue. Although it is known to be expressed in mouse and human tissues and implicated in early murine development, its role in the retina has not been established^34,35^. Among the DE anti-sense transcripts, matrix remodeling associated 8 (MXRA8), prickle planar cell polarity protein 4 (PRICKLE4) and many solute carrier family proteins among other protein coding genes were identified, which appear to be significantly downregulated at least 3 to 4-fold in AMD retina as compared to the normal.

Several anti-sense transcripts in the PRCS tissues were identified that are differentially expressed in AMD PRCS as compared to normal. The anti-sense transcription of protein coding genes EEF1A1, COL8A1, EEF2, APOD, RPE65, CLU, UBC and other ribosomal proteins responsible for eukaryotic transcription were upregulated 3 to 4-fold in the AMD PRCS as compared to PRCS (*q*-values > 1 E-15). Many noncoding transcripts, mostly belonging to the snoRNA family such as SNORD3A, SNORA73B and SNORD17, were upregulated 3 to 4-fold in AMD PRCS tissues when compared to normal PRCS tissue. The predominant anti-sense transcripts that appeared significantly downregulated (≥3 fold, *q*-value ≥ 1E-12) in AMD PRCS as compared to normal include RBP5, MST1, MYL5 and LCAT among other lincRNA, indicating that anti-sense transcription is highly varied in both retina and PRCS tissues during age-related macular degeneration.

### Differential sense expression in AMD

**Figure 6:**
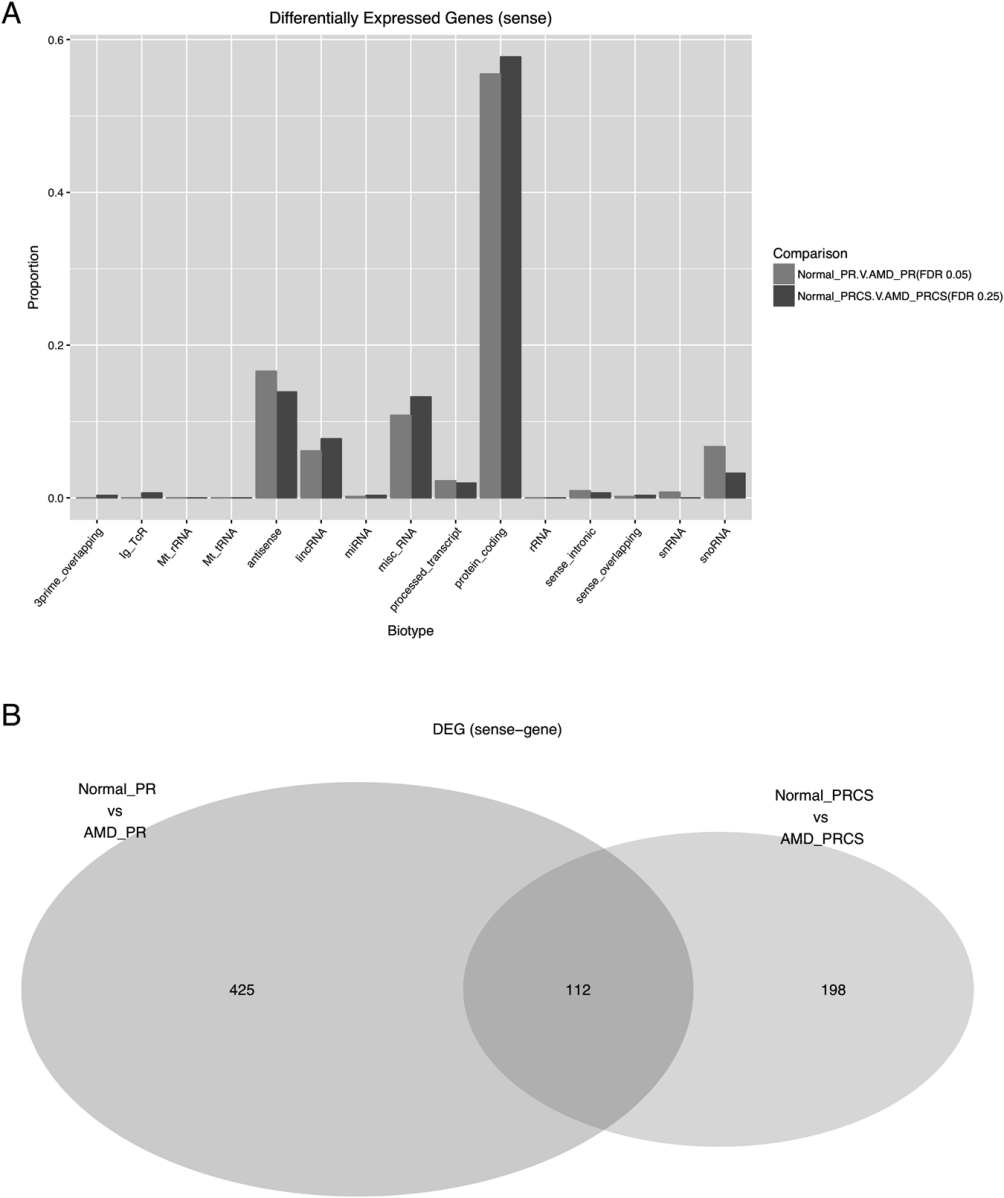
Differentially expressed sense genes between normal and AMD comparisons. (**A**) Biotypes of differentially expressed sense genes. (**B**) Venn diagram of differentially expressed sense genes between PR and PRCS.

**Figure 7:**
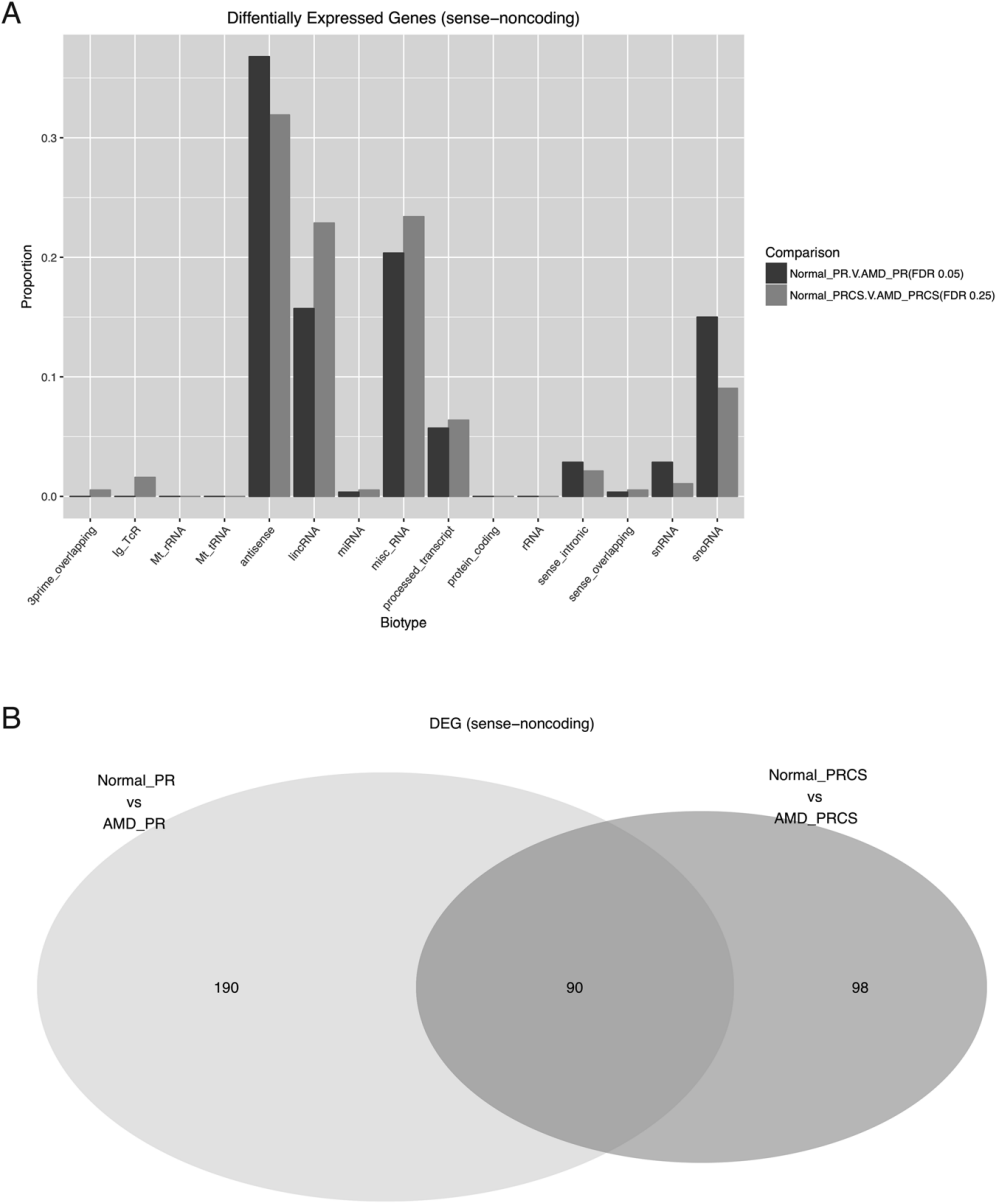
Differentially expressed noncoding genes between normal and AMD comparisons. (**A**) Biotypes of differentially expressed noncoding genes. (**B**) Venn diagram of differentially expressed noncoding genes between PR and PRCS.

For the Normal PRCS vs AMD PRCS comparison, a *q*-value cutoff of 0.25 was used, for both the sense coding genes and non-coding genes—identifying 310 (mRNA) and 188 (ncRNA) differentially expressed genes (DEGs) respectively. We conservatively expect 75% of these genes to be true positives, based on a *q*-value of 0.25. In the PR a most stringent *q*-value cutoff of 0.05 was used to identify 537 (mRNA) and 280 (ncRNA) transcripts in normal versus AMD. Supplementary Table 3 details the results of top 30 differential mRNA and ncRNA for each comparison. Figure 6 displays the expression of those DEGs across conditions. The difference between tissues was larger than the difference between disease states within the same tissue indicating strong tissue specific expression of ncRNA in these tissues. It is also interesting to note that there are transcripts differentially expressed between normal and AMD regardless of tissue. It is unclear whether this may be causal or a side-effect of AMD progression. As we are interested in the effect of disease on the noncoding landscape, we also determined the biotypes of all DE ncRNAs (Fig. 7).

### Co-localization of DE-ncRNA and pathway analysis of nearby protein-coding genes

Since most ncRNA are not well characterized and may be involved in regulation of their neighboring coding-genes, protein coding *genes in cis* to the DE ncRNAs were identified. The DE genes, ncRNA and anti-sense genes were separately investigated for their role in the different pathways that may affect the retina and PRCS function.

The majority of the noncoding RNA that was found to be differentially expressed in the AMD versus Normal PR and PRCS belonged to anti-sense transcripts (as defined by ENSEMBL) followed by small nucleolar RNA and a few lincRNA that were significantly upregulated by over 3 to 4 fold. Most of these noncoding RNA were either novel or have uncharacterized function; therefore their role in the eye has yet to be explored. The DE ncRNA from both the retinal and PRCS tissues were co-localized to genes predominantly enriched for Epithelial Adherens Junction and corticotrophin releasing hormone-signaling pathways. While the retina specific analysis identified Gap junction signaling and germ cell signaling pathways as top canonical pathways, the PRCS tissue analysis revealed remodeling of epithelial adherens junction, dermatan sulphate biosynthesis (late stages) and axonal guidance signaling as major pathways regulated by the ncRNA.

IPA analysis of the sense gene transcripts differentially expressed in the AMD versus normal retina revealed IL-22 signaling, amyloid processing, 4-1BB signaling in T lymphocytes as the top canonical pathways. Similar analysis in PRCS tissues revealed IL-22 and IL-10 signaling as the top canonical pathways along with Cardiolipin biosynthesis II and role of JAK family kinases in IL-6 type cytokines signaling pathways. The immune related pathways that were obtained in the IPA analysis in both tissues are very relevant in causing AMD pathobiology as increased circulation of complement component 5a (C5a) was observed in serum circulation of AMD patients which increases the expression of IL-17 and IL-22 cytokines^36^. It was also reported that anti-inflammatory cytokine IL-10 have both an increased accumulation of macrophages in neovascular lesions and decreased choroidal neovascularization, thus causing retinal pathology^37^.

## Discussion

A natural question arises as to the biological effect of the many genes showing highly significant anti-sense transcription consistently across all patients—a phenomenon which demands exploration. The expectation of a biological effect is strengthened by the observation that the genes showing differential anti-sense transcription are highly enriched for some pathways, in particular the EIF2 signaling pathway. The first expectation is that the anti-sense transcription affects the normal transcription of the same genes. This may be the case on a small percent of the differentially regulated genes, but the overlap between genes showing differential sense transcription and genes showing differential anti-sense transcription is small (Supplementary Fig. 2). The anti-sense transcripts could however be affecting translation and protein expression. As far as we are aware, this is the first demonstration of a consistent and dramatic global effect on antisense transcription that is associated with a disease state in humans and therefore is important information to provide the macular degeneration research community.

In this study, we present the first strand-specific comprehensive transcriptome data analysis that compares retinal and PRCS tissues isolated from human cadaver normal and AMD eye globes. Using a strand-specific RNA-Seq processing pipeline has enabled the identification of RNA transcripts produced for sense and anti-sense transcription encoded by the same genomic region. While differential expression between sense gene transcripts and ncRNA have been observed between the retina and PRCS tissues in the normal and AMD tissues, intriguing anti-sense differences were also observed. For the first time, a significant increase in anti-sense transcription in AMD eye tissues is reported—implicating a potential role in pathology of AMD. This study mainly focused on understanding the differential expression of non-coding and anti-sense transcription in PR and PRCS tissues that emerged from the RNA-Seq analysis.

Anti-sense transcription is known to regulate the gene expression either *in cis* or *in trans*, either through transcription or by association with non-coding RNA in the genome^38^. The presence of anti-sense transcripts can induce a threshold-dependent regulatory control to fine-tune gene expression. For example, it has been reported that budding yeast increases its gene expression between different cells by managing the balance between sense-anti-sense transcripts—in particular stress related genes were enriched for anti-sense transcripts^39^. This analysis could identify both sense and anti-sense regulatory mechanisms coordinated by these tissues, in a way that relates particularly to disease state.

Hierarchical clustering of our samples and diseased tissues was performed and clean separation was achieved only when anti-sense signal is used. As far as we know, the identification of significant dis-regulation of anti-sense transcription, even far beyond what is observed in sense transcription, has not been reported to date in any disease. The differential expression of anti-sense transcripts between normal and AMD retina and normal and AMD PRCS tissues revealed eIF2 regulation as the predominantly upregulated pathway in AMD tissues. The phosphorylation of eIF2alpha at Ser51 is known to inhibit the translation initiation causing a temporary shutdown of protein synthesis^40,41^. Persistent eIF2 alpha phosphorylation through regulatory kinases has been reported during stress conditions in neurodegenerative diseases like Alzheimer’s disease (AD)^42^. The phosphorylation of eIF2 is controlled by four kinases among which PKR-like endoplasmic reticulum kinase (PERK) along with GCN2, HRI and PKR^42^. Interestingly, the PERK/eIF2α/ATF4 and IRE1/ASK1/JNK cascades are the most important pathways that were associated with pathological changes like inflammation, ER and mitochondrial stress and matrix degradation in AMD^43,44^. These pathways can elicit several AMD-related pathological changes via the induction of VEGF, C/EBP homologous protein (CHOP), caspase-4 (CASP4), and nuclear factor-κB (NF-κB)^45^. The differential up regulation of eIF2 observed in AMD retina and RPE may be due to increased stress response and inflammation.

Differential anti-sense expression of key complement genes in the PRCS includes C1R, C3, CFH, which are upregulated more than two-fold in the AMD tissues when compared to the normal tissues. The anti-sense RNA for other key apolipoproteins known to play a key role in AMD, such as APOE and APOE, were also differentially expressed in AMD tissues. These genes are critical for maintaining RPE and retinal homeostasis pathways, and disruption of these processes may lead to AMD. The magnitude of differential anti-sense expression observed in our study suggests that anti-sense transcription could provide another level of gene regulation in addition to post-translational and transcription factor-mediated mechanisms.

When comparing the total DE ncRNA profiles, we observed a predominant expression of small nucleolar RNA (snoRNA) in AMD tissues when compared to normal tissues. Among the DE snoRNA, we found 3-fold higher expression of SNORA73B, SNORA54 in both retina and PRCS tissues of AMD when compared to the normal tissues, indicating that they may have a role in AMD. The DE ncRNA also identified eIF2-signaling pathways as the key pathways in IPA analysis for both the retina and PRC tissues reiterating their role in AMD.

Strict thresholds were used where possible to minimizing false positives when interpreting the differentially expressed genes, non-coding and anti-sense RNA between normal and AMD. Due to the limited RNA availability of the macular retina and PRCS tissues in the AMD donor eye samples (a region which is necessary for central vision and critical in age-related macular degeneration), this study only addressed the differential expression and transcriptome changes observed in the peripheral retina and PRCS tissues. The sample size (n = 8 donors) provided enough power to detect transcriptome level differences between the normal and AMD tissues. Nevertheless, a transcriptome analysis of a large dataset of samples with different AMD progression levels and types (early to advanced/ neovascular forms) is warranted to identify stage specific expression of DE non-coding RNA and genes that may be used as biomarkers for tracking AMD progression and pathology. This data also showed strong correlation with the previously published FPKM values of the peripheral retina and PRCS tissues^23,30^.

Pseudogenes dominated the differentially expressed transcripts in both the Retina and PRCS tissues. Many common pseudogenes were found that were differentially up- and down-regulated in both of these tissues. As their roles in ocular biology and human disease have not been fully investigated, they have been removed from this analysis, allowing a focus on the remaining signatures. This approach may have potentially eliminated some pseudogenes that may be functionally relevant during AMD.

With the advancement of next generation sequencing technologies and reduced costs, there is a promise that more eye-specific data will be available to analyze the differential non-coding transcriptome profiles in individual eye tissue layers. This will provide an opportunity to uncover novel pathways to study the pathophysiology of different eye diseases such as retinal degeneration, macular degeneration and other eye pathologies regulated by ncRNA. In summary, the present transcriptome analysis in the retina and PRCS tissues has increased our knowledge of the coding and non-coding regions of the genome expressed in these tissues. However, the exact spatial expression patterns of most of these genes and ncRNA are still unclear, as are the *in vivo* functions of these ncRNAs in retinal/ocular development and AMD pathogenesis. Functional studies of ncRNAs in the retina and other ocular tissues have the potential to greatly enrich our understanding of normal and disease processes of the eye and inspire novel therapeutic strategies.

## Materials and Methods

### Eye collection

Our study was approved by the University of Pennsylvania Institutional Review Board (IRB) and conformed to regulations for use of human subject research at University of Alabama and at University of Pennsylvania (UPenn). The eye tissues for our study were isolated from eight pairs of eyes collected from non-diabetic Caucasian donors who registered voluntarily to donate to the eye bank—with informed consent about the tissue use for research from donors with a death-to-preservation interval of <6 hr. The first set is collected from donors with mean age of 73.9 yr ± 12.5 yr (mean ± standard deviation), and, the second set was collected from donors with mean age of 84.6 ± 7.2 yr (mean ± standard deviation), to maximize the number of eyes with AMD pathology. All eyes were collected and processed by the Alabama Eye Bank recovery personnel and are preserved in RNA-later (Qiagen, Valencia, CA, USA) for the left eye and, 2% glutaraldehyde and 1% paraformaldehyde in 0.1 M phosphate buffer for the right eye^46^. The left eyes were shipped overnight on wet ice to UPenn, and were processed upon arrival. The normal or AMD status of donor right eyes was assessed by Dr. Christine A. Curcio at the University of Alabama by a three-component protocol as described previously^46^. The fellow eye design was adapted in our study following well-documented literature^47–49^. The left eyes, preserved in RNA-later solution, were examined by photography with stereo-microscope before dissection. For each donor eye, the retina (PR) and RPE-Choroid-Sclera (PRCS) samples were dissected from the peripheral region of the posterior eye globes using a 10 mm-biopsy punch followed by a 8 mm-punch in the middle of the 10 mm-punch to minimize the sample contamination. The PR was collected separately from PRCS into a 1.5 ml tube and stored separately until further processing. The age and gender of all subjects is reported in Supplementary Materials, neither of which is confounded with disease state.

### Library preparation and RNA-Seq runs

Eight normal and eight AMD donor eyes produced 16 PR and 16 PRCS samples resulting in 32 RNA assays. These were prepared using the AllPrep DNA/RNA Mini kit (Qiagen). The RNA quality was determined using R6K Screen Tape on a 2200 Tape Station (Agilent, Santa Clara, CA, USA) and was quantified using Qbit-BR (Broad Range) assay kit on a Qbit 2.0 Fluorometer (Life Technologies) following manufacturers instruction. Only RNA with integrity number (RIN) value of >8.5 was used for preparing sequencing libraries. To sequence the transcriptome, library preparation and sequencing was done using the TruSeq Stranded total RNA with RiboZero Gold kit (Illumina, CA) protocol with a total RNA (800 ng) as the starting material. A total of 32 libraries were prepared with unique barcode sets and their quality was determined using Agilent DNA1000 chip following manufactures protocol. All the DNA libraries with mean peak size of 260 bp were processed for sequencing. The libraries were sequenced on an Illumia HiSeq. 2000 machine following manufacturers protocols. A total of 16 lanes were run for sequencing (2 libraries/lane with a 100 bp Paired End reads) to achieve sequencing depth of 200 million 100-bp paired-end reads per sample (Supplementary Table 1).

### RNA-Seq quality control

The RNA-Seq data for all the samples in our study are deposited in GEO (accession number GSE99248). Pre-alignment QC showed average quality scores for both forward and reverse reads to be ≥ 30 throughout the length of transcripts. Reads were then mapped to hg19 using STAR^20^ with a mapping rate ≥ 93% for all samples which indicates high quality. Post-alignment QC revealed one normal RPE/Choroid sample with an excessively high level of rRNA, two AMD RPE samples with detectable levels of retinal contamination, and three AMD samples (two retina and one RPE samples) with high percentage of chrM expression. These six samples were therefore removed prior to further analysis (Supplementary Table 1). Two samples (TR01 and TR13) were re-sequenced due to an insufficient number of reads.

### RNA-Seq data analysis

RNA-Seq reads from each sample were aligned to hg19 using STAR version 2.5.1b^20^. Data were normalized at the read level, prior to quantification, using the PORT pipeline v0.8.2a-beta (https://github.com/itmat/Normalization). All pseudogenes were filtered from the ENSEMBL annotation (GRCh37.p13) due to unreliable alignments. The normalized SAM files were then quantified at the gene level by identifying, for each gene, all reads that were consistent with some ENSEMBL annotated splice form of the gene. Differential expression (DE) analysis was performed between each pair of tissue/disease type by computing Limma-Voom^50^ *p*-values for each gene and then performing a Benjamini-Hochberg correction for multiple testing, to produce q-values. Hierarchical clustering was based on Jensen-Shannon divergence in R^51^. Cis neighboring genes were identified using Genomic Regions Enrichment of Annotations Tool (GREAT) with default parameters for co-localization analysis^52^ with default settings and the co-localized coding genes were analyzed with Ingenuity Pathway Analysis (IPA) (Qiagen)^53^. All AMD GWAS associations were found using the NHGRI-EBI Catalog and lifted over from GRCh38 to hg19 using Ensemble’s Converter tool. Transcript biotypes were defined using the “gene biotype” information in the ENSEMBL annotation (GRCh37.p13). Those ncRNA with average depth of coverage equal to, or greater than 1 were considered expressed.

## Electronic supplementary material

Supplementary Information

Supplementary Table 1

Supplementary Table 2

Supplementary Table 3

Supplementary Table 4

Supplementary Table 5
