# Supplementary material for: Complete Transcriptome Profiling of Normal and Age-Related Macular Degeneration Eye Tissues Reveals Dysregulation of Anti-Sense Transcription
Source: Sci Rep. 2018 Feb 14;8:3040. doi: 10.1038/s41598-018-21104-7 (PMC5813239; doi:10.1038/s41598-018-21104-7)

Complete Transcriptome Profiling of Normal and Age-Related Macular Degeneration Eye Tissues Reveals Dysregulation of Anti-Sense Transcription

Eun Ji Kim, Gregory G. Grant, Anita S. Bowman, Naqi Haider, Harini V. Gudiseva,
Venkata Ramana Murthy Chavali

Supplementary Information:

Supplementary Figure 1. Differentially expressed anti-sense transcription. The coverage plots were generated merging normalized coverage of 5 replicates in each condition. The plus and minus tracks are displayed and scaled separately. Differentially expressed anti-sense expression is shown in (A) one exon gene RN7SK, (B) multi-exon gene RHO, (C) one exon gene RN7SL2 and (D) multi-exon gene EEF1A1 across all eight conditions.

Supplementary Figure 2. Overlap between differential sense and anti-sense genes. Venn diagram of differentially expressed sense and anti-genes from (A) normal PR and AMD PR comparison and (B) normal PRCS and AMD PRCS comparison.

Supplementary Table 1. Donor information and alignment metrics for all samples. For each sample, age, sex, race, tissue type, disease status (histological phenotype), total number of reads, percentage of uniquely-mapped reads, ribosomal RNA rate and chrM rate are listed.

Supplementary Table 2. Read count and differential anti-sense gene expression results. Anti-sense gene counts from each sample, p-values from limma-voom for differential expression, Benjamini-Hochberg corrected q-values.

Supplementary Table 3. Top 30 differential ncRNA and mRNA results. ncRNA and sense gene counts from each sample, p-values from limma-voom for differential expression, Benjamini-Hochberg corrected q-values of the top 30 differentially expressed genes for each comparison.

Supplementary Table 4. Expression of the marker genes. Sense gene level read counts from each sample for six marker genes (RPE65, BEST1, RHO, OPN1LW, OPN1SW, RGR) and hierarchical clustering of six marker genes across 26 samples.

Supplementary Table 5. Gene counts for RN7SK, RHO, RN7SL2, EEF1A1. Gene counts of individual samples for genes RN7SK, RHO, RN7SL2, EEF1A1. Merged coverage was shown in Figure 4 to show differentially expressed anti-sense transcription.

Supplementary Figure S1.

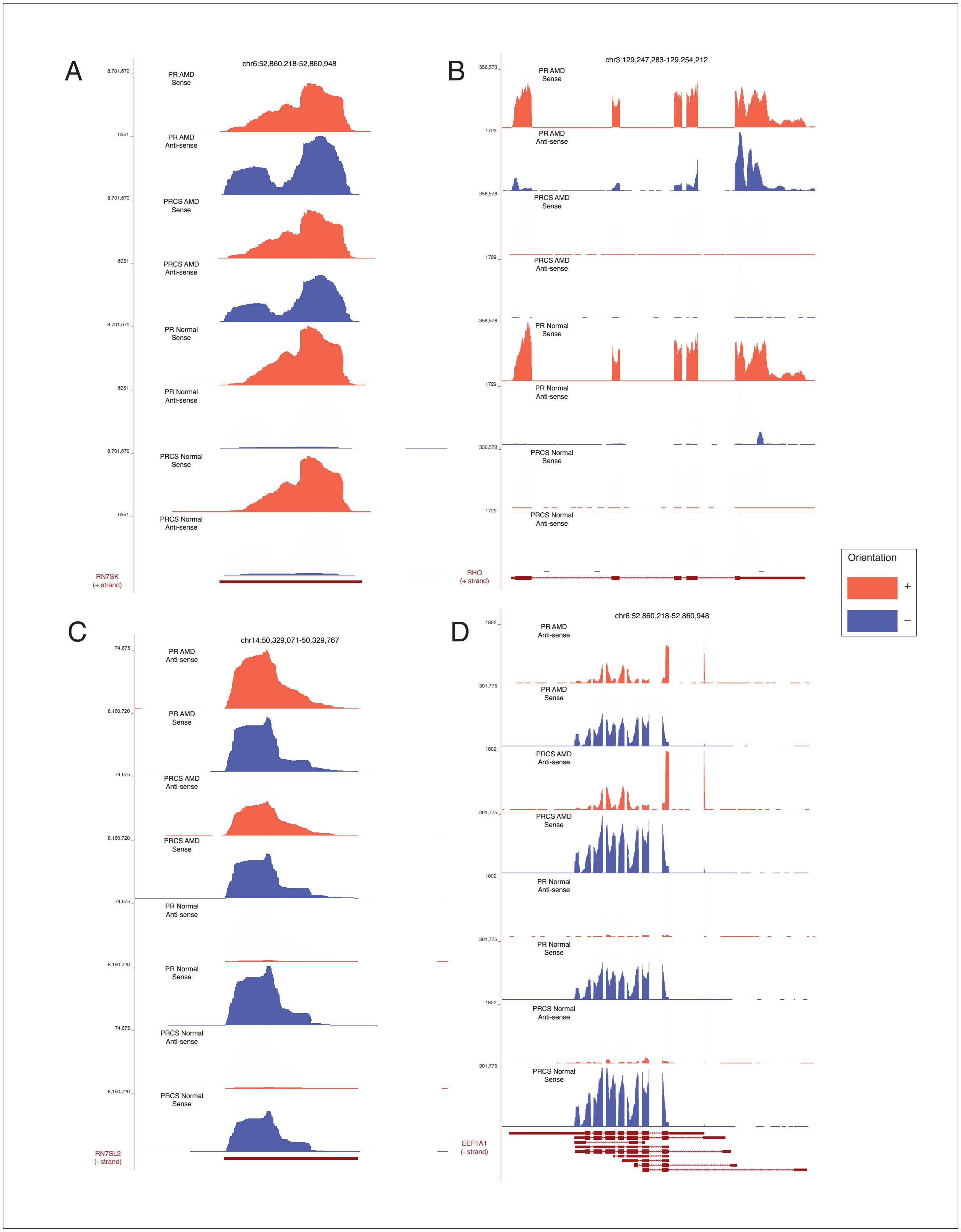

Supplementary Figure S2.

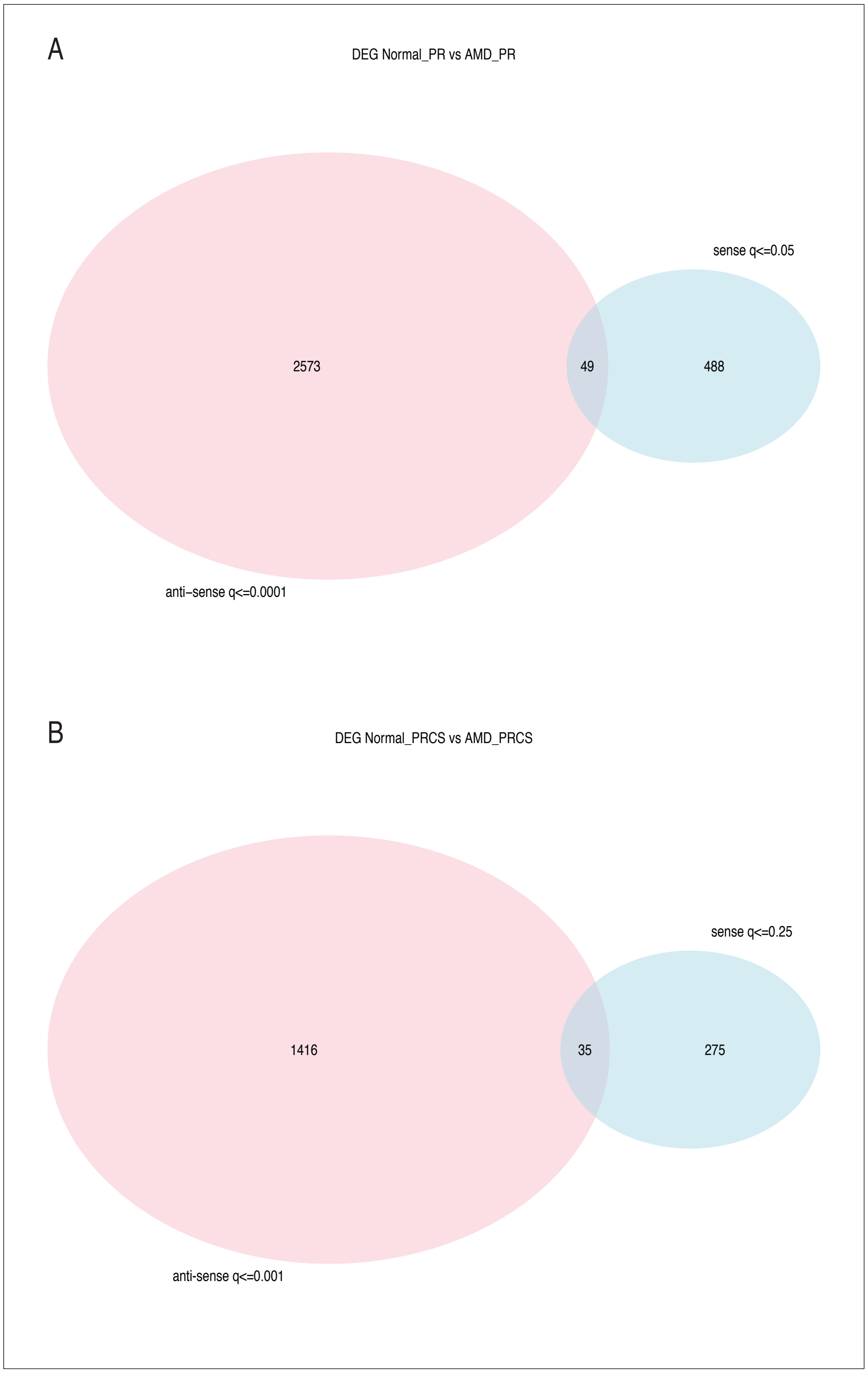

Supplement: Supplementary file 1 — Supplementary Information [file 41598_2018_21104_MOESM1_ESM.pdf]
